# Measurement of Retinal Blood Flow Using Fluorescently Labeled Red Blood Cells^1^,^2^,^3^

**DOI:** 10.1523/ENEURO.0005-15.2015

**Published:** 2015-05-12

**Authors:** Tess E. Kornfield, Eric A. Newman

**Affiliations:** Department of Neuroscience, University of Minnesota, Minneapolis, Minnesota 55455

**Keywords:** blood flow, methods, rat, red blood cell velocity, retina

## Abstract

Blood flow is a useful indicator of the metabolic state of the retina. However, accurate measurement of retinal blood flow is difficult to achieve in practice. Most existing optical techniques used for measuring blood flow require complex assumptions and calculations. We describe here a simple and direct method for calculating absolute blood flow in vessels of all sizes in the rat retina. The method relies on ultrafast confocal line scans to track the passage of fluorescently labeled red blood cells (fRBCs). The accuracy of the blood flow measurements was verified by (1) comparing blood flow calculated independently using either flux or velocity combined with diameter measurements, (2) measuring total retinal blood flow in arterioles and venules, (3) measuring blood flow at vessel branch points, and (4) measuring changes in blood flow in response to hyperoxic and hypercapnic challenge. Confocal line scans oriented parallel and diagonal to vessels were used to compute fRBC velocity and to examine velocity profiles across the width of vessels. We demonstrate that these methods provide accurate measures of absolute blood flow and velocity in retinal vessels of all sizes.

## Abstract (precis)

Accurately measuring blood flow in the retina is an important challenge, as blood flow reflects the health of retinal tissue and is disrupted in many diseases. Existing techniques for measuring blood flow are limited due to the complex assumptions and calculations required.

## Significance Statement

Accurately measuring blood flow in the retina is an important challenge, as blood flow reflects the health of retinal tissue and is disrupted in many diseases. Existing techniques for measuring blood flow are limited due to the complex assumptions and calculations required. The techniques described here use fluorescently labeled blood cells combined with ultrafast microscopy measurements to overcome these limitations. The measurement techniques were validated by several methods. Blood flow and blood velocity were assessed in blood vessels of all sizes in the rat. Accurate measurement of retinal blood flow can be used to track disease progression and inform treatment.

## Introduction

Blood flow is closely tied to the metabolic state of cells in the CNS (Attwell et al., 2010) and is disrupted in many CNS disorders including stroke and Alzheimer’s disease (Girouard and Iadecola, 2006). In the retina, blood flow is compromised in diabetic retinopathy (Garhofer et al., 2004a), glaucoma (Garhöfer et al., 2004b), and retinal vessel occlusion (Osborne et al., 2004), among others, and can lead to severe vision loss. Reductions in blood flow and blood vessel responsiveness can occur in the early stages of disease, and accurate measurement of retinal blood flow in humans and animal models can be used to track disease onset and progression and to inform treatment.

Many methods have been developed for measuring blood flow in the retina (Pournaras and Riva, 2013). Estimates of blood flow can be made by observing the deposition of large (∼15 μm) microspheres (Ahmed et al., 2001), by measuring transit time of labeled plasma, and by measuring oxygenation with functional magnetic resonance imaging (Cheng et al., 2006; De la Garza et al., 2011) or phosphorescence lifetime imaging (Shahidi et al., 2006, 2009; Wanek et al., 2011). More commonly, blood flow is computed as the product of blood velocity and vessel cross-sectional area, each measured independently. Vessel diameter can be measured from fundus images by using the retinal vessel analyzer or the scanning laser ophthalmoscope, or by using confocal line scans. Velocity can be measured with bidirectional laser Doppler velocimetry, frequency domain optical coherence tomography (FD-OCT), video analysis of fluorescently labeled red blood cells (fRBCs) or small microspheres (Rovainen et al., 1993; Lorentz et al., 2008), or line scan analysis of RBC streaks (Kleinfeld et al., 1998; Hutchinson et al., 2006; Schaffer et al., 2006; Autio et al., 2011; Kim and Filosa, 2012; Santisakultarm et al., 2012).

Noninvasive optical techniques for measuring retinal blood flow have gained favor due to their applicability to human subjects. Recent technological advances include the Canon laser Doppler blood flowmeter, a commercially available instrument that uses Doppler velocimetry and diameter measurements to calculate blood flow (Garcia et al., 2002; Guan et al., 2003; Gilmore et al., 2005; Sehi et al., 2012). FD-OCT, which computes blood flow by integrating velocity across the vessel width (Wang et al., 2007, 2009; Doblhoff-Dier et al., 2014; Leitgeb et al., 2014; Shahidi et al., 2014) is also used. However, a major drawback of most optical methods is that blood flow measurements are limited to vessels greater than ∼30 μm in diameter; smaller vessels require higher-resolution techniques.

To this end, we have developed techniques for measuring blood flow in the retinal vessels of the rat, which range from ∼4 to ∼70 μm in diameter. We measure blood flow by visualizing the passage of fRBCs. This technique is relatively noninvasive, requiring only withdrawal and reinjection of blood. Our measurements are direct; minimal computation and few assumptions are needed to calculate absolute flow. Imaging of fRBCs has been used previously to measure blood flow in small vessels where blood velocity is low and the passage of individual cells is easily imaged. Here, ultrafast confocal line scans capture the passage of high-velocity fRBCs in large retinal blood vessels. We use this technique to measure total blood flow in the retina, blood flow changes in response to functional challenge, and blood velocity profiles across the width of vessels. Absolute blood flow can be calculated easily and accurately with this technique.

## Materials and Methods

### *In vivo* preparation

Blood flow was measured in rats using an *in vivo* retinal preparation that has been described previously (Srienc et al., 2012; Kornfield and Newman, 2014). All animal procedures were performed in accordance with the regulations of the University of Minnesota Animal Care Committee. Briefly, 2- to 3-month-old, male, Long–Evans rats were anesthetized with 2% volatile isoflurane in 30% oxygen and 70% nitrogen, and tracheotomized for artificial ventilation. The femoral vein and artery were cannulated for drug injection and the measurement of blood pressure, respectively. Following surgery, isoflurane was incrementally discontinued, and anesthesia was maintained using α-chloralose (800 mg/kg bolus, 550 mg/kg/h maintenance; α-chloralose–HBC complex; catalog # C8849, Sigma-Aldrich; the molecular weight of uncomplexed α-chloralose is 10% that of α-chloralose–HBC Complex). Pupils were dilated with atropine (atropine sulfate ophthalmic solution 1%, National Drug Code 61314-303-02, Falcon Pharmaceuticals), and a contact lens was placed over the eye. Animals were secured to the stage of an upright confocal microscope (FV1000, Olympus) using a custom holder. The paralytic agent gallamine triethiodide was given (20 mg/kg bolus, 20 mg/kg/h maintenance; catalog #G8134, Sigma-Aldrich) to reduce eye movement. During experimentation, animals were ventilated at 55 breaths/min with a tidal volume of ∼3.4 ml and 2 mmHg end-expiratory pressure. A deep breath of 30 cmHg was given every ∼5 min to maintain full lung capacity. Body temperature was maintained at 37 °C. Arterial blood pressure (Pressure Monitor BP-1, World Precision Instruments), oxygen saturation (MouseOx, Starr Life Sciences), and end-tidal CO_2_ (MicroCapStar, CWE) were monitored continuously, and data were collected only when physiological parameters were within normal range (blood pressure, 95-130 mmHg; oxygen saturation, >92%, end-tidal CO_2_, 35-45 mmHg) and stable. Following experimentation, ∼1 ml of blood was withdrawn from the arterial line for *in vitro* assessment of RBCs per blood volume and the fRBC/RBC ratio. The animal was killed by injection of potassium chloride (2 mEq/kg).

### Fluorescent labeling of red blood cells

The RBC labeling procedure has been described previously (Kornfield and Newman, 2014). Briefly, blood was withdrawn from each animal at the beginning of the experiment, and RBCs were isolated and resuspended in blood plasma buffer (BPB; in mM: 128 NaCl, 15 glucose, 10 HEPES, 4.2 NaHCO3, 3 KCl, 2 MgCl2, and 1 KH2PO4, pH 7.4; Khoobehi et al., 2003). Cells were incubated for 5 min with the lipophilic dye carbocyanide 1,1´-dioctadecyl-3,3,3´,3´-tetramethylindodicarbocyanine, 4-chlorobenzenesulfonate salt solid (DiD; catalog #D-7757, Invitrogen). Cells were washed three times to removed unbound dye. Labeled cells were then reinjected into the animal at volumes appropriate for the experiment. fRBC/RBC ratios ranged from 0.1% to 1.8%. Higher ratios were used to measure velocity and flux in small vessels, and lower ratios were used to measure flux in large vessels.

### RBC flux and diameter measurements

**Figure 1:**
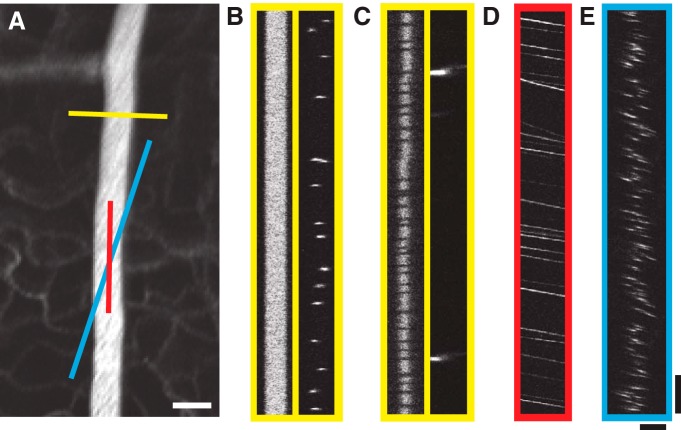
Measurement of RBC flux and velocity using confocal line scans. ***A***, A confocal reference image showing the three line scan configurations used for measuring flux and diameter (yellow line), and velocity (red and blue lines). Scale bar, 50 µm. ***B***, A line scan image of a large vessel acquired with a perpendicular line scan (yellow line in ***A***). The *y*-axis represents time, and the *x*-axis represents distance. Vessel diameter is measured by imaging FITC-labeled plasma (left), and fRBC flux is measured by imaging fRBCs (right). ***C***, A line scan image of a capillary acquired with a perpendicular line scan (yellow line in ***A***). Vessel diameter and total RBC flux is measured by imaging FITC-labeled plasma (left), and fRBC flux is measured by imaging labeled RBCs (right). ***D***, A line scan image of a large vessel acquired with a line scan parallel to the vessel (red line in ***A***). fRBC velocity is measured from the slope of fRBC streaks, where velocity is inversely proportional to the streak angle. ***E***, A line scan image of a large vessel acquired with a line scan oriented diagonal to the vessel (blue line in ***A***). fRBC velocity across the width of the vessel is measured from the slope of the fRBC streaks along the *x*-axis of the image. Scale bars in ***E*** refer to ***B–E***. Vertical calibration: ***B***, 15.5 ms; ***C***, 77.5 ms; ***D***, 18 ms; ***E***, 30.5 ms. Horizontal scale bars: ***B***, 60.5 µm; ***C***, 10.5 µm; ***D***, 38 µm; ***E***, 121.5 µm.

RBC flux and blood vessel diameter were measured and analyzed as described previously (Kornfield and Newman, 2014). Briefly, fluorescein isothiocyanate (FITC) dextran (2000 kDa; 1 ml of 3% solution; catalog #FD200S, Sigma-Aldrich) was injected intravenously for visualization of intraluminar diameter. Line scans were oriented perpendicular to the axis of the blood vessel (Fig. 1*A*, yellow line). Vessel diameter and fRBC flux were imaged simultaneously with 488 and 635 nm excitation light, respectively. Line scans in vessels <10 μm in diameter were acquired at ∼650 Hz for at least 45 s. Line scans in vessels >10 μm in diameter were acquired at 4200 Hz for 8 s (scan time was limited by the confocal microscope software). The ultrafast acquisition rate in large vessels was necessary to capture the passage of high-velocity RBCs. Diameter was extracted from FITC line scans (Fig. 1 *B*,*C*) using custom MATLAB software that automatically measured the distance between vessel borders. fRBC flux was computed as cells per second by identifying and counting bright spots in the line scan images (Fig. 1*C*).

### Velocity measurements

Blood velocity was measured with line scans oriented either parallel (Fig. 1*A*, red line) or diagonal (Fig. 1*A*, blue line) to the blood vessel. In velocity line scan images (Fig. 1*D*,*E*), fRBCs appear as diagonal streaks where the angle of the streak is inversely proportional to velocity (Dirnagl et al., 1992). Measurement of streak angle was performed using the Radon function, and the open source code for velocity calculation (Chhatbar and Kara, 2013) was modified to fit the current dataset. For diagonal line scan data, velocity was calculated at 10 locations along the length of the line. Vessel diameter was calculated from reference images of the FITC-filled vessel lumen. Due to chromatic aberration of the rat lens, the 488 and 635 images did not align perfectly. For this reason, all data are aligned to the furthest edges of the vessel that yielded usable velocity data.

### Functional challenge

Hyperoxia was generated by increasing the oxygen content of the ventilation gas from 30% to 60% and reducing nitrogen accordingly. Hypercapnia was generated by replacing 5% of the nitrogen with CO_2_. End-tidal CO_2_ reached ∼60 mmHg during hypercapnia. Blood pressure remained within the normal range during both interventions. Both blood flow and blood vessel diameter were measured in primary arterioles approximately every 30 s during the challenge sessions, which typically lasted 13 min. Baseline diameter and flow values were collected for at least 2 min prior to challenge onset. Hyperoxia and hypercapnia were maintained for at least 3 min, after which inhalation gas mixtures were returned to normal, and flow and diameter recovery were tracked.

### Calculation of fRBC/RBC ratio and measurement of RBCs per blood volume

The fRBC/RBC ratio was calculated using both *in vivo* flow cytometry and a slide-based cell-counting method. To calculate the fRBC/RBC ratio using *in vivo* flow cytometry, total RBC flux and fRBC flux were acquired simultaneously with perpendicular line scans across capillaries, where blood cells move single file (Fig.1*C*). Labeled and total cells per second were counted, and their ratio was calculated.

The fRBC/RBC ratio and the number of cells per blood volume were also calculated using a hemocytometer (catalog #3102, Hausser Scientific). Blood withdrawn from the rat at the end of each experiment was diluted 1:300 with BPB and loaded onto an unlined two-chamber hemocytometer. An upright fluorescence microscope with a 20× water-immersion objective was used to visualize labeled and unlabeled cells. Ten to 20 fields were imaged in each of the two chambers of the hemocytometer. Filter cubes of 488 and 635 nm were used to visualize RBCs and fRBCs, respectively. Using a custom MATLAB algorithm, these images were intensity rescaled, median filtered, and background subtracted. The circular RBCs in each channel were identified and counted. Each imaged field of the hemocytometer represents a defined volume, so these cell counts were used to calculate the number of cells per volume as well as the fRBC/RBC ratio. Values from the two chambers did not differ, and the data were pooled.

#### Statistics

Data are expressed as the mean ± SD. Significance was defined as *p* < 0.05. Two-tailed *t* tests were used to compare the fRBC/RBC ratios calculated from *in vivo* flow cytometry and *in vitro* cell counting. Pearson linear correlations were used to quantify the relationship between blood flow measurements calculated two ways, and to compare flow rates in parent and daughter vessels at bifurcations. Repeated-measures ANOVAs were used to assess changes in diameter and flow in response to hyperoxic and hypercapnic challenge. Statistical analysis was performed using GraphPad Prism (version 5) and MATLAB.

## Results

We have measured blood flow in rat retinal blood vessels by introducing fluorescently labeled red blood cells into the vasculature (Fig. 1). fRBC flux was measured with perpendicularly oriented confocal line scans in vessels ranging from primary arterioles and venules to capillaries by counting individual fRBCs as they passed through a vessel (Fig. 1*A*, yellow line, B,C). In small vessels where RBCs move single file, fRBC flux and total RBC flux can be captured simultaneously (Fig. 1*C*). RBC velocity was measured within vessels with confocal line scan lines oriented parallel to the vessel lumen (Fig. 1*A*, red line, D). Velocity was also measured across the full width of blood vessels with diagonally oriented line scan lines (Fig. 1*A*, blue line, E).

### fRBC/RBC ratio and RBCs per blood volume measured *in vivo* and *in vitro*

The methodology described here allows for the measurement of absolute blood flow, expressed in microliters per minute. Blood flow measurements obtained by counting fRBCs require knowledge of the fRBC flux, the fRBC/RBC ratio, and the number of RBCs per volume of blood. The fRBC/RBC ratio was calculated using *in vivo* flow cytometry by simultaneously counting fRBCs and unlabeled RBCs passing single file through a capillary (Fig. 1*C*) for at least 2 min. The fRBC/RBC ratio was also calculated from *ex vivo* counts made with a hemocytometer. The fRBC/RBC ratio measured *in vivo* equaled the ratio measured *ex vivo* in three of four experiments [*in vivo* vs *in vitro*: 1.64 ± 0.35% (*n* = 8) vs 1.52 ± 0.35% (*n* = 9); 1.50 ± 0.42% (*n* = 9) vs 1.52 ± 0.60% (*n* = 16); 0.69 ± 0.24% (*n* = 9) vs 0.71 ± 0.35% (*n* = 32); *p* = n.s.]. The experiment where *in vivo* and *in vitro* values were not statistically equal was one with a very low fRBC/RBC ratio [0.53 ± 0.36% (*n* = 8) vs 0.22 ± 0.22% (*n* = 36); *p* < 0.01, two-tailed *t* tests]. fRBC/RBC ratios ranged from 0.1% to 1.8%.

The number of RBCs per microliter of blood was calculated for each experiment from the total number of RBCs in the hemocytometer, which has a known volume. In our experiments, the mean number of RBCs per microliter was 9.6 ± 1.1 million (*n* = 15), fitting within the accepted range of 7-10 million RBCs/µl.

### Blood flow calculated from RBC flux and velocity

Blood flow (*Q*) was calculated by two independent methods that were then compared, allowing us to validate the measurements of diameter, flux, and velocity. In the first method, fRBC flux was used to compute blood flow with the following equation:

Q=(fRBCs/s) * (RBCs/fRBCs)/(RBCs/vol),

where the number of fRBCs per second was determined from line scans perpendicular to blood vessels, and RBC/fRBC and RBC/volume ratios were both determined from hemocytometer measurements. In the second method, RBC velocity and vessel diameter were measured using confocal line scans parallel and perpendicular to blood vessels, respectively. Blood flow was calculated from the following equation:

Q=V * A

where *V* is velocity, and *A* is the luminal cross-sectional area. *V* was measured from luminal line scans in the center of blood vessels, where the slope of fRBC streaks is inversely proportional to velocity. As discussed below, this yielded velocities that approximated the average RBC velocity within the vessel.

**Figure 2:**
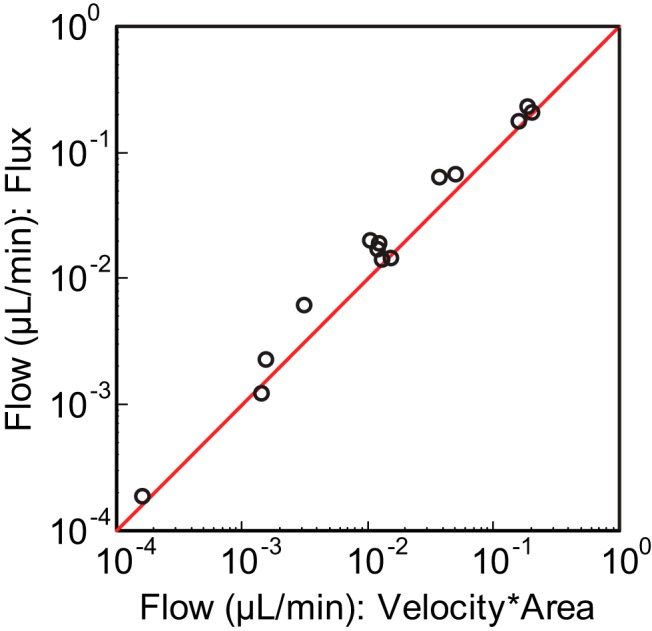
Blood flow calculated using two independent methods. Flow is calculated from fRBC flux (*y*-axis), and velocity and vessel cross-sectional area (*x*-axis). Each data point represents a single vessel. The red line represents unity.

Blood flow was calculated by these two methods in vessels with a range of diameters and flow rates. The two methods yielded blood flow values that were highly correlated (Fig. 2; *R* ^2^ = 0.982; *p* < 0.001; *n* = 15; Pearson linear correlation) with a slope deviating from unity by only 0.13. Because fRBC flux, blood velocity, blood vessel diameter, and blood cell counts are all measured independently, the highly correlative relationship between the flow values confirms the validity of our methods.

### Blood flow at bifurcations

**Figure 3:**
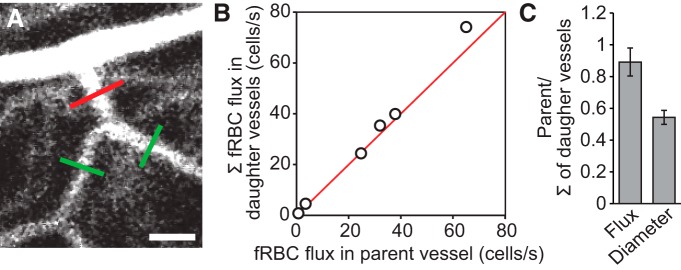
RBC flux and diameter at vessel bifurcations. ***A***, Confocal reference image showing a typical bifurcation. The red line represents the confocal line scan across the parent vessel, and the green lines represent the line scans across the two daughter vessels. Scale bar, 40 µm. ***B***, The mean RBC flux for the parent vessel is plotted against the sum of RBC fluxes in the daughter vessels for six bifurcating vessels. The red line represents unity. ***C***, The ratio of the parent to the sum of daughter vessels for RBC flux and vessel diameter.

We measured fRBC flux in parent and daughter vessels at bifurcations (Fig. 3*A*) to further validate the blood flow measurement technique. The sum of the RBC flux in the two daughter vessels should equal the flux in the parent vessel. This technique has been used previously to validate blood flux measurements (Parthasarathi et al., 1999). For all bifurcations studied, the sum of the fluxes in the daughter vessels was nearly identical to that of the parent vessel (Fig. 3*B*). This was true for vessels with a range of fluxes. The parent and daughter fluxes were highly correlated (Fig. 3*B*; *R* ^2^ = 0.995, *p* < 0.001; *n* = 6; Pearson linear correlation) with a slope deviating from unity by only 0.12. The ratio of the flux in the parent vessel to the sum of the fluxes in the two daughter branches was 0.89 (Fig. 3*C*). Parent vessels averaged 9.3 ± 3.5 μm, and daughter vessels averaged 8.4 ± 2.9 μm in diameter. The ratio of the diameter of the parent vessel to the sum of the diameters of the daughter vessels was 0.54 (Fig. 3*C*).

### Total retinal blood flow

**Figure 4:**
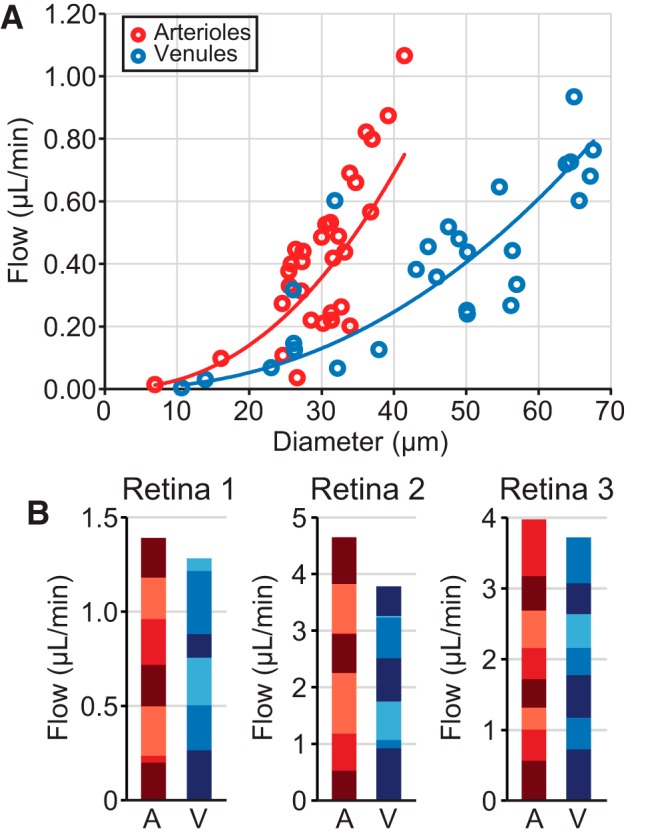
Total retinal blood flow. ***A***, Blood flow in individual primary arterioles (red) and venules (blue) emerging from the optic disk, plotted against vessel diameter and fit with power functions (see text for details). ***B***, Total blood flow in three retinas. Left columns represent arterioles (A), and right columns represent venules (V). Each color block indicates the flow in an individual vessel. The full bar height is the sum of all individual flows, corresponding to the total blood flow in arterioles or venules in a retina.

Total retinal blood flow was measured by summing the flow in all primary arterioles or venules emerging from the optic disk. Flow in these primary vessels was predictably dependent upon their diameter and type (Fig. 4*A*). The flow in individual primary retinal arterioles and venules averaged 0.42 ± 0.25 and 0.40 ± 0.25 µl/min, respectively. The diameter of the arterioles and venules averaged 30.0 ± 6.7 and 46.5 ± 16.5 μm (*n* = 4 retinas, 32 arterioles and 29 venules). The ratio of the number of arterioles to venules in this sample was 1.10. The relationship between flow and diameter can be described roughly by power functions with exponents of 2.30 (*R* ^2^ = 0.65) and 2.23 (*R* ^2^ = 0.79), respectively, for arterioles and venules. These values are similar to the exponents of 2.76 and 2.84, respectively, reported previously for the human retina (Riva et al., 1985).

Because all blood that enters the retina through arterioles must leave it through venules, we expected that total blood flow in arterioles would equal the total blood flow in venules. To calculate the total blood flow in these vessels, we summed the individual flows of all arterioles and venules in the retina. Arteriole flow averaged 3.34 µl/min, while venule flow averaged 2.93 µl/min. The ratio of arteriole/venule blood flow was 1.13 for the three retinas examined (Fig. 4*B*).

Total retinal blood flow, calculated as the average of arteriole and venule flows, was 1.34, 4.22, and 3.85 µl/min for the three retinas examined, with a mean of 3.14 µl/min. A large variation in total blood flow between individuals has also been observed in human retinas (Garhofer et al., 2012; Tayyari et al., 2014).

### Functional challenge

We challenged animals with hyperoxia and hypercapnia, two stimuli known to affect retinal blood flow, to confirm that our measurements are sensitive to changes in blood flow. Diameter and blood flow were measured in primary arterioles before, during, and after functional challenge.

**Figure 5:**
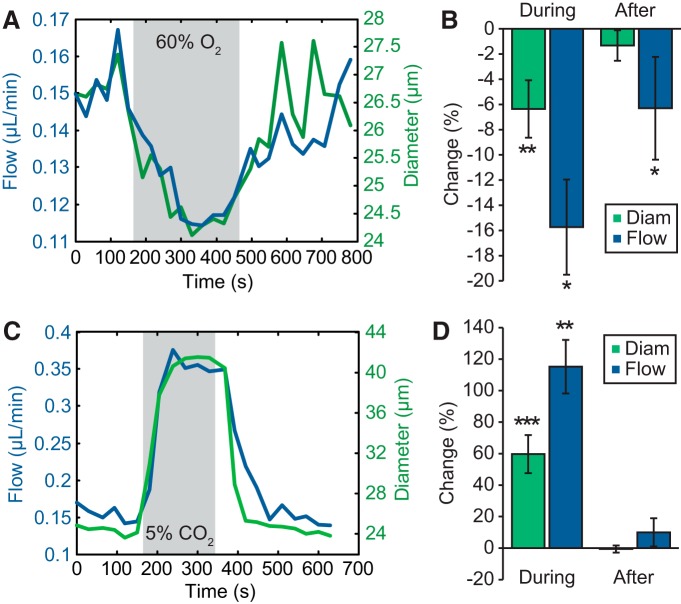
Blood flow changes in primary arterioles in response to hyperoxic and hypercapnic challenge. ***A***, ***C***, Representative traces of flow (blue) and diameter (green) in response to hyperoxia (***A***) and hypercapnia (***C***). The period of hyperoxic or hypercapnic challenge is indicated by gray shading. ***B***, ***D***, Percentage change in diameter and flow during and after the challenge period. *p < 0.05, **p < 0.01, ***p < 0.001. *n* = 4 for hyperoxic challenge and *n* = 3 for hypercapnic challenge. Diam, Diameter.

Hyperoxia, generated by raising inhaled O_2_ from 30% to 60%, constricted vessels and decreased blood flow (Fig. 5*A*). Arteriole constrictions averaged 6.4 ± 2.3%, a significant reduction from baseline diameters (*p* < 0.01, repeated-measures ANOVA; *n* = 4), and blood flow decreased by an average of 15 ± 3.8% from baseline (Fig. 5*B*; *p* < 0.05). The changes in diameter and blood flow had a slow onset, taking ∼170 s to reach steady-state values. Following the return to normoxia, diameter and flux moved toward baseline, although only diameter fully returned to baseline within the 5 min recovery period.

Hypercapnia, generated by breathing 5% CO_2_, triggered a dilation of 59.7 ± 12.1% and a blood flow increase of 115.2 ± 17.0% (Fig. 5*C*,*D*), values that were greater than baseline levels (*p* < 0.001 and *p* < 0.01, respectively; *n* = 3). The blood flow changes were faster than for hyperoxia, taking ∼45 seconds to peak. Diameter and flow values at the end of the recovery period were indistinguishable from baseline values.

### Velocity profile across the width of vessels

In theory, blood velocity in rigid, straight, nonbranching vessels containing a homogenous fluid should follow a parabolic relation across the width of the vessel, with velocity maximal at the vessel center and approaching zero at the edges. A perfect parabolic relation does not typically occur in blood vessels *in vivo*, but quadratic relations are used to approximate blood velocity across the width of vessels as small as ∼15 μm. We used two different approaches to measure velocity profiles of retinal arterioles and venules.

#### Parallel line scans

**Figure 6:**
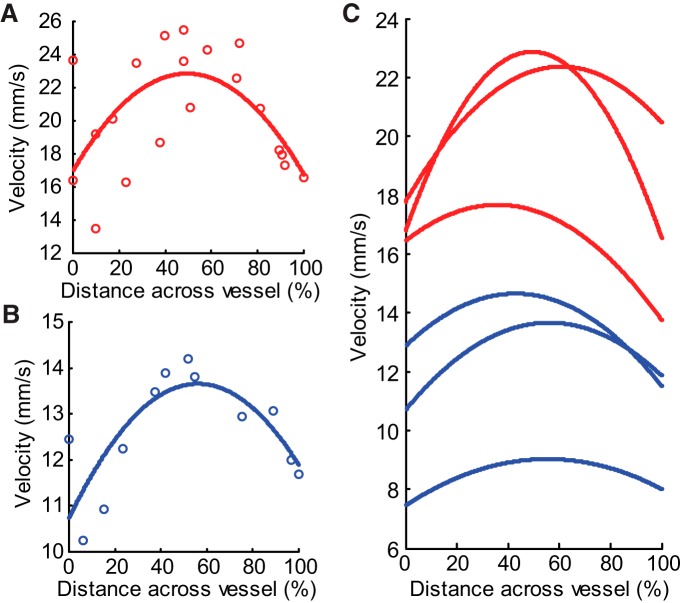
RBC velocity profiles across the width of blood vessels using parallel line scans. ***A***, ***B***, Velocity was determined at many points across the width of an arteriole (***A***) and a venule (***B***) by positioning the line scan at different points within the vessel lumen. Velocity was calculated from the slope of fRBC streaks in line scan images (see text for details). Velocity measurements were fit with a second-order polynomial (solid lines). ***C***, Parabolic fits of RBC velocities for individual arterioles (red) and venules (blue).

High-frequency line scans parallel to the vessel lumen were acquired at multiple locations across the width of the vessel. These line scan images contain angled streaks created by fRBCs as they move through the vessel (Fig. 1*D*). Shallower angles indicate higher velocity. For each set of line scans in a single vessel, the fRBC velocity was plotted as a function of the position across the width of the blood vessel. The dataset for each vessel was fit with a quadratic equation. Raw data and the parabolic fit for a representative arteriole and venule are shown in Figure 6, *A* and *B*. As expected, velocities were lowest at the vessel edges and highest in the center of the vessel (Fig. 6*C*). The mean maximum velocities were 21.0 mm/s for arterioles with an average diameter of 28.4 μm, and 12.4 mm/s for venules with an average diameter of 35.7 μm.

#### Diagonal line scans

**Figure 7:**
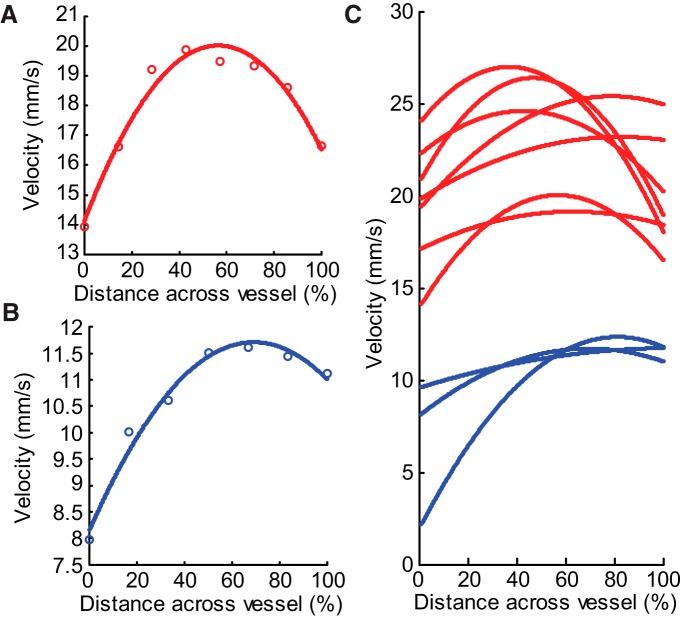
RBC velocity profiles across the width of blood vessels using diagonal line scans. ***A***, ***B***, Velocity was determined at many points across the width of an arteriole (***A***) and a venule (***B***) by measuring the angle of fRBC streaks along the diagonal line scan. Velocity measurements were fit with a second-order polynomial (solid lines). ***C***, Parabolic fits of RBC velocities for individual arterioles (red) and venules (blue).

A second method was used to measure velocity profiles in single trials. High-frequency line scans oriented diagonally across a vessel (Fig. 1*E*) captured the velocity of individual fRBCs across the vessel width. Velocity was computed at 10 positions along the line. The advantage of this method is that the full velocity profile can be determined from a single line scan trial. Examples of velocity profiles from an arteriole and venule are shown in Figure 7, *A* and *B*. Fitting the data from each trial with a quadratic equation yielded an average maximum velocity of 23.7 mm/s for arterioles and 11.9 mm/s for venules (Fig. 7*C*).

Although we did not measure velocity profiles in a comprehensive sample of arterioles and venules, the velocity values and parabolic shapes of the profiles were similar between those obtained with parallel and diagonal line scans.

## Discussion

We describe and characterize a technique to measure blood flow in rat retinal vessels. By capturing the passage of individual fRBCs through vessels, and by using a simple method of adjusting fRBC flux to account for the fRBC/RBC ratio and RBCs per volume, absolute blood flow was calculated in vessels ranging from 4 to 70 μm in diameter. The accuracy of these measurements was tested a number of different ways. First, blood flow measurements based on fRBC flux were compared to a second independent method of calculating blood flow based on fRBC velocity and vessel diameter. Second, we tested whether blood flow in parent and daughter vessels at bifurcations matched expected values. Third, we observed blood flow changes in response to hypercapnic and hyperoxic challenges. Finally, parabolic velocity profiles were evident in blood vessels as small as 20 μm in diameter.

### Blood flow at bifurcations

The validity of blood flux measurements was tested at vessel bifurcations, where the sum of blood flow in daughter branches is equal to flow in the parent vessel. Conservation of blood flow was indeed observed, providing assurance that our method accurately captured the passage of individual RBCs in different caliber vessels. The sum of diameters of daughter vessels did not equal the diameter of the parent vessel. Murray’s law predicts vessel diameters required for maximum energetic efficiency at branch points. Theoretically, the radii of parent (*r_p_*) and daughter (*r_d1_* and *r_d2_*) vessels follows a power law, where *r_p_^k^* = *r_d1_^k^* + *r_d2_^k^* with the exponent *k* ranging from 2 to 3. This was not observed in our bifurcation dataset, where *k* averaged 6.8. A study of the cerebral vasculature shows a wide variability of *k* values (Cassot et al., 2009), with the current result close to their reported mean of 6.2. Our results confirm previous findings that Murray’s law does not accurately model vessel diameters at bifurcations, particularly in microvessels (Cassot et al., 2009; Hirsch et al., 2012).

### Total retinal blood flow

We have used fRBC flux measurements to determine blood flow in all primary arterioles and venules, providing proof of principle that this simple method can be used to calculate total blood flow in the retinal vasculature. Our blood flow estimate of 3.14 µl/min is on the low end of published values for the rat retina. Those values range from 2.1 µl/min using fluorescently labeled blood cells (Khoobehi et al., 2003) to 6.3 µl/min using OCT (Srinivasan and Radhakrishnan, 2013), and 12.6 µl/min using microsphere tracking (Leskova et al., 2013; Shih et al., 2014). Anesthesia has a strong effect on blood flow. In an OCT study, retinal blood flow was 3.3 µl/min under ketamine/xylazine and 6.4 ml/min under isoflurane/xylazine (Choi et al., 2012). Isoflurane is a known vasodilator (Franceschini et al., 2010).

The blood flow we measured can also be expressed as 0.42 ml/min/g, using an average retina weight of 7.5 mg (Shih et al., 2014). This blood flow value is similar to previous measurements made in rabbit (Xuan et al., 1999), cat (Roth and Pietrzyk, 1994; Ahmed et al., 2001), and mouse (Muir et al., 2012), although differences in measurement technique and anesthesia limit the value of these comparisons (Schmetterer and Kiel, 2012). We also noted a consistent blood flow underestimation of 12.2% in venules compared with arterioles. The discrepancy is likely a systematic measurement error. Venules have larger diameters than arterioles, and some fRBCs within venules may have been out of the plane of focus and too dim to be detected.

### Functional challenge

The vessel diameter and blood flow changes we observed in response to hyperoxia and hypercapnia were similar to published values (Kiss et al., 2002; Sato et al., 2003; Gilmore et al., 2005; Venkataraman et al., 2006; Sehi et al., 2012; Werkmeister et al., 2014), and the time course of the blood flow changes seen were comparable to those previously observed in the rat (Hétu et al., 2013). The Hagen-Poiseuille relation predicts that under constant perfusion pressure, blood flow should vary as the fourth power of vessel diameter. In practice, blood flow deviates from this ideal relation. In previous studies, hypercapnia and hyperoxia produced blood flow changes that were smaller (Sato et al., 2003) or larger (Chung et al., 1999; Kiss et al., 2002; Gilmore et al., 2005) than the predicted change. The blood flow changes we observed during hypercapnia and hyperoxia were smaller than predicted by the fourth power relation. The reason for this discrepancy is unclear. Hyperoxic and hypercapnic challenge will have wide systemic effects that could lead to significant changes in perfusion pressure at the vessels being monitored.

### Comparison to other methods of measuring blood flow

Variations of the fRBC imaging method have been used over the past 30 years to monitor blood flow (Sarelius and Duling, 1982; Zimmerhackl et al., 1983; Unthank et al., 1993; Collins et al., 1998; Schulte et al., 2003; Wright and Harris, 2008; Kornfield and Newman, 2014). fRBC imaging has been used to measure capillary flux and velocity by tracking the movement of labeled cells in video frames (Ben-Nun et al., 1992; Yamaguchi et al., 1992; Hudetz et al., 1995; Ben-Nun, 1996; Parthasarathi et al., 1999; Seylaz et al., 1999; Krolo and Hudetz, 2000; Schulte et al., 2003; Reyes-Aldasoro et al., 2008; Wright and Harris, 2008; Wang et al., 2011). With advances in imaging technology, fRBCs have also been tracked with confocal microscopy (Villringer et al., 1994; Pinard et al., 2002; Tomita et al., 2011) and scanning laser ophthalmoscopy (Wajer et al., 2000). A technique for computing absolute blood flow in human retinas by imaging fRBCs with scanning laser ophthalmoscopy has been patented (U.S. Patent US6554775 B1). In the current study, we combined numerous existing methodological techniques with high-speed confocal line scans, allowing us to measure absolute blood flow in large vessels as well as capillaries, and affording significant insights into blood flow dynamics in the retina.

Several groups have developed sophisticated techniques for measuring components of blood flow without cell labeling. Laser scanning microscopy can generate 3D maps of velocity and flux in normal and tumorous brain tissue (Kamoun et al., 2010), an approach particularly valuable in tissues with 3D vessel structures. Cross-correlation algorithms can extract velocity and flow data from high-speed video recordings in rat muscle preparations (Ortiz et al., 2014), and RBC velocity can be measured in human retinal capillaries using scanning laser ophthalmoscopy (Bedggood and Metha, 2012). Calculating blood flow from unlabeled blood is less invasive than using fRBCs, and the methodologies cited above benefit from high temporal and spatial resolution. The strength of our method lies in its comparative simplicity: it can be used with any confocal microscope system with line-scanning capabilities and uses relatively simple analysis procedures. Further, our method can extract multiple parameters (flux, velocity, and diameter) from a single acquisition and can be used in concert with other blood flow measurement techniques including laser speckle flowmetry (Srienc et al., 2010).

Our approach to measuring absolute blood flow has distinct advantages over other optical methods that are typically applied in human retinas. Measurements based on bidirectional laser Doppler velocimetry, the retinal vessel analyzer, and OCT are limited to vessels >30 μm in diameter and are at their most accurate in large-diameter vessels. FD-OCT applied in the rat retina cannot measure blood flow in vessels <30 μm (Werkmeister et al., 2014). In contrast, our technique can be used to calculate flow in retinal vessels of all sizes in the rat retina. Unlike other optical methods of measuring blood flow, which require sophisticated calculations to extract flow values, our technique is straightforward. It is based on simple *in vivo* and *in vitro* cell counts, which we have shown to be reliable and accurate.

No gold standard technique exists for measuring retinal blood flow in animals or humans. However, the approach we describe can be applied easily and inexpensively, and can provide quantitative blood flow values for vessels of all sizes. Unlike most previous techniques for measuring fRBC flux, which detect changes in flux during stimulation, the present method combines existing techniques to calculate absolute flow with millisecond resolution. Our technique can be applied to other species and can be used to quantify blood flow change in both normal and pathological retinas.

### Methodological limitations

One limitation of our method is that it uses RBC flux as a proxy for blood flow. Calculated values of blood flow in individual vessels will not perfectly represent the flow of whole blood, which also comprises plasma, white blood cells, and other particulate matter. Therefore, care must be taken when using this method to compare flows between vessels. Hematocrit is heterogeneous in individual vessels and varies most in capillaries (Pries et al., 1995; Ganesan et al., 2010; Gould and Linninger, 2015), where the coefficient of variation is 0.60 (compared to 0.41 in arterioles and venules; Pries et al., 1995). Further, the Fahraeus effect, defined as the decrease in hematocrit that accompanies decreasing vessel diameter, could make direct comparisons of blood flow between vessels of different sizes difficult (Barbee and Cokelet, 1971). However, in our dataset, estimates of blood flow based on RBC velocity and vessel diameter (which do not depend on hematocrit) yielded values very close to those calculated from RBC flux across the entire vascular network (Fig. 2), indicating that systematic hematocrit variations did not measurably alter our ability to accurately measure blood flow.

Although the difficulties that arise from hematocrit differences should be kept in mind, many currently employed methodologies rely on RBC flux to calculate blood flow and are subject to the same limitations as our techniques. Plasma-borne fluorescent tracers can be used to measure blood flow, an approach that avoids the problem of inhomogeneous hematocrit by measuring plasma flow instead of particulate flow. However, these angiographic techniques have limitations of their own (Schmetterer and Garhofer, 2007). The dye dilution technique, which tracks the transit time of injected fluorescein through the circulation, relies on a number of assumptions (Pournaras and Riva, 2013), including the assumption that all of the blood entering a retinal region from a primary arteriole exits that region from the corresponding venule. Schmetterer and Garhofer (2007) conclude that retinal blood flow measurements based on fluorescein angiography may not be reliable, particularly under pathological conditions. In addition, angiography techniques do not capture the transit time of the quantal unit of oxygen delivery, the red blood cell (Baker et al., 1980; Tomic et al., 2001). Tomic et al (2001) directly compared blood flow measurements in the monkey retina made using fluorescein angiograms and labeled microspheres. They found that there was only a weak correlation between blood flow values measured by the two techniques and concluded that mean transit time measurements made using angiography do not accurately measure retinal blood flow.

Another limitation of our method is that it uses confocal line scans, which can only be performed on one vessel at a time if high temporal resolution is to be preserved. Because the technique involves blood draws and reinjection of labeled blood cells, it is unlikely to be favored in human subjects over less invasive, though also less direct, techniques for measuring blood flow. However, the technique is particularly useful in animal studies, where flow can be measured in all vessels, regardless of size, and invasiveness is not a concern.

### Parabolic velocity profiles

We measured the velocity of fRBCs using confocal line scans to determine velocity profiles across the width of primary arterioles and venules. The profiles of vessels between 20 and 50 μm in diameter were roughly parabolic. The two methods used to capture velocity profiles, parallel and diagonal line scans, yielded similar data. Diagonal line scans require only a single trial to sample the entire profile and are therefore not subject to intertrial shifts in velocity, making them preferable to parallel scans. The advantages and limitations of using angled line scans have been discussed previously (Kamoun et al., 2010). The maximum velocities for arterioles and venules obtained in this study match published values well (Doblhoff-Dier et al., 2014), although they are below velocities measured using fluorescent microspheres (Lorentz et al., 2008).

The parabolic velocity profiles we obtained resemble those reported for larger vessels but do not perfectly match theoretical estimates. There are a number of reasons for this. First, we cannot capture the maximum fRBC velocity within a vessel due to the relatively large depth of field of our imaging system. Velocity measurements in the center of a vessel in the *x*–*y* plane will include velocity values from RBCs at the edges of the vessel in the *z*-plane. Therefore, our estimates of maximum velocity are underestimated and approximate the average fRBC velocity. This will cause the resulting quadratic fits to be flatter than expected. Other rheological factors also contribute to blunting at the apex of the parabolic blood velocity profiles, an observation reported previously (Rovainen et al., 1993; Parthasarathi et al., 1999; Logean et al., 2003; Zhong et al., 2011). Second, although some studies detect zero velocity at vessel edges using Doppler-based systems (Yazdanfar et al., 2003; Zhi et al., 2011), we did not. This is not surprising for vessels with diameters ranging from 20 to 50 μm, where the width of a single RBC represents a significant fraction of the vessel diameter. As vessel diameter decreases, individual RBCs take up a proportionally larger area of the lumen, and their interactions with each other and the vessel wall disrupt laminar flow (Zhong et al., 2011).

### Conclusions

Our results demonstrate that blood flow can be calculated simply and accurately in vessels from 4 to 70 μm in diameter by imaging fRBCs with ultrafast line scans, and scaling those counts using the number of RBCs and fRBCs per volume of blood. Flow estimates obtained from this procedure match those calculated from independent measurements of RBC velocity and vessel diameter. Flow at vessel bifurcations confirms the accuracy of the technique. Measurements of total blood flow in the retina and velocity profiles across the width of vessels are easily obtained using fRBC tracking. The method will prove useful in future studies of blood flow and metabolism in the retina and throughout the CNS.

## Synthesis

The decision was a result of the Reviewing Editor Maiken Nedergaard and the peer reviewers coming together and discussing their recommendations until a consensus was reached. A fact-based synthesis statement explaining their decision and outlining what is needed to prepare a revision is listed below.

Synthesis of Reviews: this is potential a nice study, but additional experiments and discussion of the literature is needed.

Reviewer #1

The goal of this study was to develop techniques for precise measurement of blood flow in retinal vessels. The approach involved the study of anesthetized paralyzed rats and visualizing the passage of fRBCs in the retina. Along with baseline data, changes in blood flow to two stimuli (hypercapnia and hyperoxia) were tested. From the data, the authors conclude that the method they describe provides precise and accurate measures of absolute blood flow and velocity in retinal vessels of all sizes. While some elements of the study appear to be new, there are several issues with this study and its presentation.

1. The paper repeatedly states that absolute blood flow was measured. In reality, the description indicates that blood flow was calculated based on other variables that were directly measured. Please be more precise throughout.

2. What are the references listed in the text as: Anonymous, 2012, 2014?

3. Recordings of blood pressure were included in the study, a positive. Along these lines, how stable was blood pressure during the actual measurements of diameter and velocity? Was it affected by interventions like hypercapnia?

4. The main conclusion regarding resting blood flow was based on only an n of 3, and the values are quite variable. For reasons that are not well justified, the authors state this was expected. Variability could be due to a number of factors, and a larger n is typically needed for a new method to be rigourously evaluated and convincing. The authors have not performed a comparison between the current new approach and any other established (gold standard) approach in the same animals. Related to this point, the paper does not provide any kind of serious discussion of how resting values obtained in this study compare with the literature. There are many previous studies that have quantified retinal blood flow (using microspheres and other approaches) in multiple species (eg, rat, cat, rabbit, monkey, pig, etc). For whole tissue and when expressed on a per weight basis, how do flow values in the current and previous studies compare?

5. The authors conclude that the method used here to obtain blood flow is precise (has high precision). This statement is also not entirely convincing. For example, the changes in blood flow are not close to what one would predict based on the diameter changes that are shown. With hypercapnia for example, the change in blood flow is much much less than would be expected based on the diameter change shown (Fig 5). The authors state this was expected because of perfusion pressure changes that likely occurred, but they provide no evidence for such changes or effects in this model. An alternative (that has not been excluded to this point) is that the method for obtaining values for blood flow in the current study is not as precise as is being concluded.

6. How do you interpret the changes in venous diameter? Are these changes active or passive?

7. The manuscript stated that PCO2 was 35-45 mmHg, but only end-tidal CO2 measurements were mentioned. It also says sO2 was greater than 92 mmHg, but states elsewhere that O2 saturation was measured. For both CO2 and sO2, values are presented in mmHg, but it is unclear how that value was derived based on what was measured? During hypercapnia, what level of PCO2 was produced?

Reviewer #2

The presented work documents the use of fluorescent red blood cell monitoring by video microscopy as an approach to assessing retinal blood flow. The approach would appear to provide practically useful information that could be relevant in the context of experimental studies. A few questions arise.

1) It would seem important to emphasize that the methodology provides information only about red cell transit. Issues relating to hematocrit variations within microvasculature have received considerable attention (e.g., Gould and Linninger, Microcirculation 22:1-18, 2015), and any limitations this may place on the interpretation of the results should be considered.

2) There has also been recently published work using video imaging of unlabeled red cells (Ortiz et al., Physiol. Meas. 35:351-367, 2014), so it would seem relevant to compare the limitations and advantages of these approaches.

3) As a very minor point, it might be better to state anesthetic dose in terms of the alpha-chloralose content of the administered complex (10% of total) to permit more direct comparison with past studies.

## Author Response

Dear Dr. Nedergaard,

As suggested in your letter of 4 February, we have revised our manuscript, “Measurement of retinal blood flow using fluorescently labeled red blood cells.” In all instances, we have followed the advice of the reviewers in revising the manuscript. We thank the reviewers for their helpful comments, which have greatly strengthened the paper. We have made the following changes to the manuscript.

Reviewer #1

The reviewer asks that we be more precise when referring to the method we used in calculating blood flow. To address this, we have made a number of changes to the text to indicate that blood flow was not measured directly but was calculated from multiple measurements.

References that refer to our previous work were listed as Anonymous. This was required by the eNeuro submission guidelines as a way to preserve the double-blind nature of the review process. References to our previous work remain listed as Anonymous in the revised manuscript.

The reviewer asks about the stability of blood pressure in our experimental animals. Blood pressure remained within the normal range (95 - 130 mmHg) during experimentation and did not vary greatly over time. This has been clarified in the manuscript (lines 95-97). Blood pressure was not tracked in real time during hyperoxia and hypercapnia but it remained within the normal range. This is noted in the revised manuscript (lines 141-142).

We thank the reviewer for pointing out that our measurements of resting blood flow are not adequately discussed in the context of the existing literature. We have revised the manuscript to include a more thorough discussion of this issue, comparing our flow values with those from previously published studies (lines 339-347). Our measurement of total retinal blood flow serves as proof of principle, not as a comprehensive analysis of blood flow in the rat. Thus, a large sample size is not necessary. This is clarified in the text (lines 338-341). In addition, as suggested by the reviewer, we have added a paragraph comparing our measurements of blood flow, expressed per unit weight of tissue, to previous studies (lines 346-354).

The reviewer points out that our blood flow measurements may not be precise, particularly as demonstrated in the hypercapnia experiment showing less flow change than expected for a given change in vessel diameter. We have revised the manuscript to provide a more complete explanation of this observation (lines 361-369). In addition, we have removed in all instances the word “precise” from the manuscript.

All evaluations of venous blood flow were done under baseline conditions. The hyperoxic and hypercapnic challenge data came from arterioles only. The manuscript has been revised to clarify this (lines 143, 267, 774).

We thank the reviewer for pointing out errors in documenting blood gas parameters. Indeed, we measured end tidal CO_2_ and not pCO_2_, and sO_2_ and not pO_2_. The manuscript has been revised to correct these errors (line 97). The pCO_2_ value recorded during hypercapnia has also been added (line 141).

Reviewer #2

The reviewer rightly suggests that we should more thoroughly discuss the impact of local hematocrit on our results. The manuscript has been revised to include a discussion of this issue (lines 407-413).

We thank the reviewer for calling to our attention the paper by Ortiz et al. In the revised manuscript, we compare this and other image-processing methods to our own (lines 385-396).

We agree with the reviewer that chloralose dosing can be confusing and have clarified the dosing in the manuscript to better allow for comparison between studies (lines 84-86).
